# Effectiveness of the Positive Nursing Practice Environment Promotion Programme in improving patient safety in primary health care: a study protocol for a randomised controlled clinical trial

**DOI:** 10.3399/BJGPO.2025.0025

**Published:** 2025-12-19

**Authors:** Soraia Cristina de Abreu Pereira, Eduardo José Ferreira Santos, Cintia Silva Fassarella, Olga Maria Pimenta Lopes Ribeiro

**Affiliations:** 1 Abel Salazar Biomedical Sciences Institute, University of Porto, Porto, Portugal; 2 Nursing School of the University of Porto, Porto, Portugal; 3 RISE-Health, Porto, Portugal; 4 Polytechnic Institute of Viseu, Higher School of Health, Viseu, Portugal; 5 Health Sciences Research Unit-Nursing (UICISA: E), Coimbra, Portugal; 6 Evidence-Based Practice Center of Portugal (PCEBP): A JBI Centre of Excellence, Coimbra, Portugal; 7 Faculty of Nursing, The State University of Rio de Janeiro, Rio de Janeiro, Brazil; 8 Nursing School of the University of Porto, Porto, Portugal

**Keywords:** working conditions, patient safety, primary health care

## Abstract

**Background:**

In the past decade, interest in researching nursing practice environments has increased considerably. Multiple studies have highlighted that substantial benefits result from enhancing these environments. A strong association has been established between the nursing practice environment and key factors such as professional satisfaction, safety climate, staff retention, and the quality and safety of care delivered.

**Aim:**

To evaluate the effectiveness of the Positive Nursing Practice Environment Promotion Programme (PPAPEP) in improving patient safety in primary health care.

**Design & setting:**

A randomised clinical trial will compare changes in nurses' perceptions of the safety climate and nursing practice environment in primary healthcare units. The sample will include at least 34 nurses participating in the programme and currently working in primary healthcare units. The sociodemographic and professional characteristics of the participants will be analysed, and stratified randomisation will be conducted.

**Method:**

The intervention group will participate in the PPAPEP, consisting of six training sessions, each lasting 3 hours. The programme’s goal is to empower nurses by providing knowledge about what constitutes a positive nursing practice environment and equipping them with tools to improve their practice environment. The outcomes of the intervention will be assessed both at the end of the programme and 3 months after its conclusion.

**Conclusion:**

We anticipate that this study will provide valuable insights into the effectiveness of a capacity-building programme targeted at nurses and its impact on their perceptions regarding the safety climate and nursing practice environment.

## How this fits in

Positive nursing environments play a crucial role in enhancing care quality, safety, and professional wellbeing. The Positive Nursing Practice Environments Promotion Programme (PPAPEP) is a validated programme to improve nursing practice environments. Conducting a clinical trial to assess the effectiveness of PPAPEP is essential to understand its impact on the safety climate and practice environments. The results of this study may provide definitive and reliable evidence for strategies and future nursing practice improvements, and may support the development of institutional policies to improve nursing care.

## Introduction

Nursing practice environments (NPE) have gained significant interest owing to their impact on patient outcomes, professional wellbeing, and institutional performance.^1–3^ Communication and collaboration among professionals are vital for patient safety, reducing errors, and adverse events.^4–6^ Additionally, the NPE plays a crucial role in shaping clients’ perceptions of care quality, with positive environments associated with increased client satisfaction regarding care safety.^5,7–10^ A positive NPE is also linked to improved nurse motivation, satisfaction, and retention.^11–17^ Workplaces with adequate resources, balanced workloads, and meaningful professional recognition reduce stress, burnout, and the intention to leave the profession, while ensuring greater professional safety and wellbeing.^18–24^ NPE also influence institutional indicators, improving health outcomes, pain management, and reducing falls, infections, sepsis, and pressure ulcers.^5,7,25,26^ Additionally, they mitigate absenteeism and turnover, improving institutional productivity and efficiency.^19,23^

Given this broad range of outcomes associated with positive NPE, it is essential to define this specific concept. A *'positive nursing practice environment'* refers to a workplace that facilitates the delivery of high-quality care, supports healthcare professionals, encourages their involvement in decision making, and promotes effective communication and collaboration.^27^ Effective communication, collaboration, trust, and transparency between healthcare professionals, patients, and families are essential for reducing errors and adverse events.^28,29^ A favourable safety climate, well-established safety policies, and a secure environment for professionals help minimise conflict-induced errors, promote the safety of both patients and healthcare professionals, and create a positive cycle that enhances care quality, client satisfaction, institutional success, and nurses' wellbeing.^23,30–32^

Considering the relevance of NPE and their broad impact, developing and implementing strategies to improve these environments is essential.^4,19^ The literature identifies various strategies designed to improve aspects of nurses' work environments; however, these are often isolated measures.^18^ Given the scarcity of broad strategies addressing multiple domains of the NPE, we developed Positive Nursing Practice Environments Promotion Programme (PPAPEP), a multicomponent programme designed to promote nurses’ education on what constitutes a positive NPE and provide them with tools to improve their work environment. It was developed based on characteristics or variables influencing NPE, the attributes of positive NPE, and their associated outcomes. This programme was validated through a Delphi study, in which experts recognised in the field assessed and refined the programme.^33^ The programme’s contents are presented in Table 1.

**Table 1.** Content of the PPAPEP

Session	Content
1)	Presentation of the PPAPEP. Characteristics and variables related to clients and professionals that contribute to a positive nursing practice environment
2)	Characteristics and variables related to institutions that contribute to a positive nursing practice environment
3)	Attributes of a positive nursing practice environment
4)	Outcomes of a positive nursing practice environment related to professionals
5)	Outcomes of a positive nursing practice environment related to institutions and clients
6)	Group dynamics to consolidate the knowledge acquired throughout the PPAPEP. Conclusion of the PPAPEP

PPAPEP = Positive Nursing Practice Environments Promotion Programme

The final version of the programme, improved through expert input, comprises six sessions that focus on essential themes, including factors influencing clients and healthcare professionals, organisational dimensions, the features of a positive NPE, and its outcomes.^33^ The high participation rates and positive responses from the expert panel highlight the programme’s significance and potential impact. The programme’s active-learning approach facilitates reflection and discussion, enhancing communication, teamwork, and collaboration. Delivered through presentations, videos, and interactive exercises, PPAPEP is designed to enhance work environments and perceptions of safety climate. This randomised controlled trial (RCT) aims to evaluate the effectiveness of the PPAPEP in enhancing the perceptions of primary healthcare nurses regarding safety climate and the NPE. It represents the first RCT to test the PPAPEP, potentially strengthening the evidence base on the relationship between the work environment and professionals' perceived safety climate. Additionally, it seeks to provide evidence of the programme’s effectiveness in promoting a positive NPE, guiding institutional strategies, policies, and future research.

## Method

### Study design and settings

This randomised, controlled, and parallel clinical trial will be conducted with investigator and participant blinding (double-blinding). Participant recruitment took place between September and October 2024 in a group of health centres within a local health unit in northern Portugal. This protocol follows the Standard Protocol Items: Recommendations for Interventional Trials (SPIRIT) guidelines^34,35^ and the extension for reporting results in clinical trial protocols.^36^ Additionally, the trial will adhere to the Consolidated Standards of Reporting Trials (CONSORT) guidelines for clinical trials.^37^ The study has been registered on the ClinicalTrials.gov platform with the ID: NCT06762015.^38^

### Hypotheses

This study aims to test the following two main hypotheses:

- the PPAPEP improves nurses' perceptions of the safety climate in primary healthcare units; and
- the PPAPEP enhances nurses’ perceptions of their NPE in primary healthcare settings.

### Study participants

The study will include all nurses working in primary healthcare units, such as family health units, primary healthcare centres, among others, who express interest in the programme, agree to participate in the study, regardless of professional category or experience or academic qualifications. Participants who give informed consent but fail to attend all six sessions will be excluded from the study.

### Participant selection, recruitment, and consent

**Figure 1.**
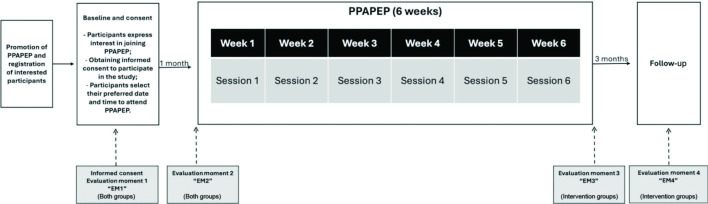
Study design and workflow. PPAPEP = Positive Nursing Practice Environments Promotion Programme

Participants will be recruited through the institution’s training centre. The training centre coordinator will send all nurses employed at the institution information about the programme, along with a link to the informed consent form, a registration form, and a declaration of interest in participating. Each participant’s declaration of interest will be coded using an algorithm provided by the research team, ensuring data anonymisation and preventing the investigator from identifying participant allocation. Study design and workflow is presented in Figure 1.

### Participant retention strategies

To minimise dropouts and improve participant retention, a schedule with the dates and times of each session will be sent to all participants, allowing them to choose their preferred session day and time in advance.

### Sample size

Considering the study’s objective, we calculated the sample size using G*Power software (version 3.1.9.6) with a two-tailed test to compare two dependent means (Student’s *t*-test). The parameters used included an effect size of 0.5, a significance level (α) of 0.05, and a statistical power (1-β) of 0.80. The calculation suggests a minimum total sample size of 34 participants (17 participants per group). The allocation ratio between the groups will be 1:1. Thus, we aim to recruit at least 34 nurses working in primary healthcare units who are interested in and agree to participate in the study.

### Allocation, randomisation, and blinding

Participants will be allocated into two groups (intervention and control) and stratified randomisation will be performed to ensure homogeneity between the groups (1:1). Stratification will consider participants’ self-assigned codes along with sociodemographic and professional characteristics such as sex, age, educational background, professional category, total professional experience, experience in primary health care, and the type of unit where they work. Stratified randomisation will be executed using RANDOM.ORG by an independent researcher uninvolved in the PPAPEP intervention or data analysis, because it offers true random numbers that are for many purposes better than the pseudo-random number algorithms typically used in traditional computer statistical software. Regarding the randomisation during each evaluation point, questionnaires will be completed by participants, sealed in opaque envelopes labelled with the respective evaluation moment ('EM0', 'EM1', 'EM2, or 'EM3'). After the final evaluation moment ('EM3'), the questionnaires will be selected based on the administration timeline illustrated in Figure 1. This approach enables participants to choose their preferred day and time for sessions without compromising group homogeneity or randomisation. It also minimises selection and information bias by ensuring that neither the researcher nor the participants are aware of their allocation until data collection is complete. Assessments during the intervention were conducted using self-administered questionnaires, and participants were instructed not to share session information with others.

### Intervention group

The intervention group will participate in the PPAPEP, which will be delivered in-person by a trained member of the research team in a designated room within the study site. The evaluations for this group will occur at pre-intervention ('EM0'), post-intervention ('EM2'), and 3 months' post-programme ('EM3'), as shown in Figure 1.

### Control intervention

For the control group, evaluations will be conducted at pre-intervention ('EM0') and before the intervention sessions ('EM1'). Although they will eventually participate in the programme, only evaluations preceding the intervention will be considered.

### Outcomes evaluation

Participants will complete a sociodemographic questionnaire, and the programme’s effectiveness will be assessed using two instruments: the shortened version of the Scale for the Environments Evaluation of Professional Nursing Practice (SEE-Nursing Practice)^39^ and Safety Attitudes Questionnaire (SAQ) — Short Form.^40^

The SEE-Nursing Practice consists of 59 items across three subscales: structure (29 items); process (19 items); and outcome (11 items). Participants rate each item on a five-point Likert scale (1: never to 5: always). Higher scores indicate a more favourable NPE for care quality and professional wellbeing. Scores are interpreted as follows:<35% (unfavourable), 35–55% (moderately favourable), 55–75% (favourable), and>75% (very favourable).^39^

The SAQ — Short Form 2006 assesses healthcare professionals' perceptions of patient safety attitudes. It has been previously validated and culturally adapted to the context in which the study will be conducted.^40^ The SAQ — Short Form 2006 comprises six dimensions, addressing organisational factors, work environment factors, and team factors, with a total of 30 items. Participants respond to each item using a five-point Likert scale ('strongly disagree' to 'strongly agree'). The final score ranges from 0–100, where 0 represents the worst perception and 100 the best perception of the safety climate.^40^ The timing of the outcome measures and data collection evaluation moments are detailed in Table 2, following SPIRIT guidelines and the extension for reporting trial protocol outcomes.^36^

**Table 2.** Outcome measures and data collection evaluation moments

Activity	Study period					
	T – 1 (Pre-allocation)	EM0 (baseline)	EM1 (pre-intervention)	Intervention period	EM2 (post-intervention)	EM3 (follow-up)
Enrollment						
- Participants' information	X					
- Participants express interest in PPAPEP	X					
- Participant consent	X					
- Random group allocation		X				
Intervention: PPAPEP			—	—	—	—
Outcomes						
Data collection:						
- Demographic and professional data	X					
- Scale for the Environment's Evaluation of Professional Nursing Practice — Shortened Version		X	X		X	X
- Safety Attitudes Questionnaire — Short Form		X	X		X	X
- Qualitative data from participants’ knowledge during intervention				X		

EM = evaluation moment. PPAPEP = Positive Nursing Practice Environments Promotion Programme

### Data analysis

Following the CONSORT guidelines,^37^ a flowchart outlining participants’ progression throughout the study will be created. This will include information on sample size, reasons for exclusions, and participant dropouts.

Data analysis will be performed by the research team, with preliminary steps including verification of data discrepancies, potential biases, anomalies, incomplete or missing data, as well as assessments of distribution normality, linearity, and homoscedasticity. These steps ensure compliance with prerequisites for subsequent statistical analyses.

Demographic and professional characteristics of participants will be analysed using descriptive statistics. This will involve measures of central tendency, variance metrics, frequency charts, and 95% confidence intervals for all subgroups.

The analysis will include group comparison tests appropriate for the type of variable under study. A univariate analysis will assess each variable before and after the intervention. Bivariate analyses will examine correlations between dependent and independent variables, while multivariate analyses will explore interactions and relationships among them.

Primary outcomes will focus on participants’ perceptions of the NPE and safety climate in primary healthcare settings. The Student’s *t*-test will be used to compare group mean values, and the paired *t*-test will examine changes within groups. Effect size interpretations will use Cohen’s D index, categorised as small (0.2–0.49), moderate (0.5–0.8), or large (>0.8).^41^

Multivariate analysis will include linear regression for dependent variables such as nurses' perceptions of the practice environment and safety climate in primary health care. If assumptions of normality, homoscedasticity, and variable independence are not met, variable transformation will be considered. After regression analysis, the adequacy of the model in representing the observed data will be evaluated, along with the possibility of changes in variable effects.^42^

### Data security and management

Questionnaires will remain anonymous, and data analysed in aggregate form and access restricted to the research team. Each participant will create a unique and confidential code to link pre- and post-intervention responses, ensuring anonymity. The research team will securely store the database with password protection, accessible only to investigators.

### Test monitoring

All assessment instruments used for participant evaluation have been translated and validated for the study population. Pretest questionnaires were administered to nurses to identify and address potential errors or inaccuracies and to evaluate response times.

## Discussion

### Summary

The study evaluates a training intervention for primary healthcare nurses using a rigorous methodological design. Given the scarcity of clinical trials in this area, the intervention is expected to empower professionals, encourage reflection on the work environment, and improve their wellbeing. In addition to providing evidence of the programme’s effectiveness, the study aims to guide institutional policies, inspire future research, and contribute to healthier work environments, positively impacting job satisfaction, professional retention, and the quality of patient care.

### Strengths and limitations

Results from this study will be reported in accordance with CONSORT guidelines and disseminated through peer-reviewed scientific journals and conferences focused on this field. Despite efforts to minimise bias, it is possible that nurses expressing interest in this training programme already have prior awareness of the topic, potentially influencing their perceptions of the NPE and patient safety (this may represent some level of performance bias). Some participants may be unable to attend all sessions owing to professional or personal reasons; to prevent this bias (dropout bias), sessions will be offered on two different days and at varied times. This study marks a significant advancement in the exploration of NPE, not only by analysing the outcomes of a training intervention for primary healthcare nurses, but also through its rigorous study design.

### Implication for research and practice

This study aims to provide robust evidence of the programme’s effectiveness in fostering positive NPE, informing institutional strategies, guiding evidence-based policies development, and inspiring future research for healthier, more supportive, and productive workplaces. These outcomes may enhance not only nurses’ professional satisfaction, but also retention and the quality and safety of patient care.

## Clinical Trial registration number

NCT06762015
